# Incorporating Evolutionary Information and Functional Domains for Identifying RNA Splicing Factors in Humans

**DOI:** 10.1371/journal.pone.0027567

**Published:** 2011-11-16

**Authors:** Justin Bo-Kai Hsu, Neil Arvin Bretaña, Tzong-Yi Lee, Hsien-Da Huang

**Affiliations:** 1 Institute of Bioinformatics and Systems Biology, National Chiao Tung University, Hsin-Chu, Taiwan; 2 Department of Computer Science and Engineering, Yuan Ze University, Taoyuan, Taiwan; 3 Department of Biological Science and Technology, National Chiao Tung University, Hsin-Chu, Taiwan; 4 Core Facility for Structural Bioinformatics, National Chiao Tung University, Hsin-Chu, Taiwan; Inserm U869, France

## Abstract

Regulation of pre-mRNA splicing is achieved through the interaction of RNA sequence elements and a variety of RNA-splicing related proteins (splicing factors). The splicing machinery in humans is not yet fully elucidated, partly because splicing factors in humans have not been exhaustively identified. Furthermore, experimental methods for splicing factor identification are time-consuming and lab-intensive. Although many computational methods have been proposed for the identification of RNA-binding proteins, there exists no development that focuses on the identification of RNA-splicing related proteins so far. Therefore, we are motivated to design a method that focuses on the identification of human splicing factors using experimentally verified splicing factors. The investigation of amino acid composition reveals that there are remarkable differences between splicing factors and non-splicing proteins. A support vector machine (SVM) is utilized to construct a predictive model, and the five-fold cross-validation evaluation indicates that the SVM model trained with amino acid composition could provide a promising accuracy (80.22%). Another basic feature, amino acid dipeptide composition, is also examined to yield a similar predictive performance to amino acid composition. In addition, this work presents that the incorporation of evolutionary information and domain information could improve the predictive performance. The constructed models have been demonstrated to effectively classify (73.65% accuracy) an independent data set of human splicing factors. The result of independent testing indicates that *in silico* identification could be a feasible means of conducting preliminary analyses of splicing factors and significantly reducing the number of potential targets that require further *in vivo* or *in vitro* confirmation.

## Introduction

Alternative splicing (AS), in eukaryotes, is one of the mechanisms of post-transcriptional regulation that generate multiple transcripts from the same gene. These transcripts are then translated into multiple proteins having diverse biological functions. According to the comparative alignment of EST sequences and high-throughput biotechnology techniques such as exon/exon-junction array and RNA-Seq, it has been revealed that most genes (larger than 90%) undergo alternative splicing in humans [1], [2], [3], [4]. In general, alternative splicing is regulated by splicing factors (SF) that recognize and associate with specific RNA sequence elements in order to enhance or repress the ability of the spliceosome to recognize nearby splice sites [5], [6]. More precisely, the mechanism is finished through many of the positive or negative trans-acting splicing factors which are recruited to the enhancer or silencer cis-acting sequence elements of the pre-mRNA, such as exonic splicing enhancer (ESE), exonic splicing silencer (ESS), intronic splicing enhancer (ISE) and intronic splicing silencer (ISS) [7], [8], [9]. Meanwhile, the process exploits the dynamic composition of splicing factors under various cell lines or developmental stages to have flexible intermolecular interactions such as protein-RNA, RNA-RNA, and protein-protein interactions [10], [11], [12]. Cancer cells often take advantage of this flexibility to produce proteins that promote growth and survival [13].

Eukaryotic messenger RNAs (mRNAs) are produced by accurately removing introns from precursors (pre-mRNAs) in a process called RNA splicing. RNA splicing is required for typical eukaryotes that produce mature mRNA before it can be used to code a correct protein through translation. The eukaryotic RNA splicing is done in a series of reactions that are catalyzed by the spliceosome, which is a collection of small nuclear RNAs (snRNAs) and proteins recruited to pre-mRNAs for carrying out intron excision [14], [15]. With the comprehensively biochemical and genetic studies in a variety of biological systems, spliceosomes have been revealed to contain five essential snRNAs, each of which functions as an RNA–protein complex called a small nuclear ribonucleoprotein (snRNP) [16], [17]. RNAs and proteins cooperate extensively in ribonucleoproteins (RNPs) to bring about the biological functions of splicing machinery [11]. Two types of spliceosomes have been identified for eukaryotes: one is U2-type spliceosome, which consists of U1, U2, U4, U5, and U6 snRNPs; the other is U12-type spliceosome, which is composed of U11, U12, U4atac, U5, and U6atac snRNPs [16]. The U2-type spliceosome catalyzes the removal of most introns and U12-type spliceosome recognizes less than 1% of human introns [18].

Regulation of pre-mRNA splicing is achieved through the interaction of RNA sequence elements and a variety of RNA-splicing related proteins (splicing factors) [19], [20]. Within the assembled spliceosome, intron excision contains two major chemical steps: the first step refers to the 5′ splice site cleavage and lariat formation; the second step refers to the 3′ splice site cleavage and exon ligation [14]. The initial event of RNA splicing is the recognition of specific sequences located at the 5′ and 3′ splice sites by splicing factors [21], which determines the intron boundaries. One of the well-known protein families of splicing factors in terms of serine- and arginine-rich carboxy-terminal domains is the SR proteins. This protein family consists of at least five different proteins with molecular masses of 20, 30, 40, 55, and 75 kD [15]. However, although the introns are excised with a high degree of precision, the splice site sequences are weakly conserved [16], [22]. The alternative selection of splice sites (alternative splicing) present within a pre-mRNA, leads to the production of multiple mRNAs from a single gene [13].

Due to the multiplicity of protein–protein and protein–RNA interactions that modulate the associations between splicing factors and pre-mRNAs, the first mass spectrometry-based analysis of in vitro-derived spliceosomes was limited to species visible in stained 2D-gels. This analysis was able to identify 17 previously known splicing factors (including hnRNP proteins) and 23 novel splicing-related proteins [23]. Although previous works have identified more than 200 human splicing factors based on comprehensive proteomic analysis [24], [25], many of the newly identified proteins have not yet been experimentally verified to function in pre-mRNA splicing [16]. Without functional validation, it would be premature to label all of these proteins as bona fide splicing factors. A previous work by Jurica and Moore [14] have manually conducted about 180 human splicing factors by literature survey.

Due to the importance of splicing factors in pre-mRNA splicing, more attention is being paid to mass spectrometry-based proteomic studies [19], [24], [25], [26], [27], which has been observed to identify an increasing number of experimentally verified splicing factors. However, experimental identification is proven to be time-consuming and lab-intensive. Thus, *in silico* investigation has the potential for characterizing splicing factors prior to experimental verification. Over the last few years, several studies have been proposed to computationally predict RNA-binding proteins [28], [29]. Additionally, many computational methods have been developed to identify RNA-binding residues on protein sequences [30], [31], [32], [33], [34], [35], [36], [37], [38], [39]. In particular, SFmap [21], a web server for predicting putative splicing factor binding sites in genomic data, utilizes a modified Hamming distance formula to define a match between a splicing factor sequence query and a target sequence. The distance scores are then standardized and a Z-score is obtained for calculating the significance of each query relative to a background model which is then compared to a threshold value in order to give a probable prediction. Another work done by Barbosa-Morais *et al.* [16] presents a semi-automated computational pipeline to aid in identifying and annotating spliceosomal proteins. The proposed method utilizes annotated human splicing factors grouped into families based on full-length homology, functional domain, and Ensembl protein family classification which are then transformed into phylogenetic trees. Their work has revealed more than 200 proteins of multiple organisms for which there is experimental evidence regarding its involvement in splicing. Furthermore, a related work by Zheng *et al.* [40] proposed a method which utilizes support vector machine, a binary-class classification algorithm, to construct a model for discriminating transcription factors (TFs) from non-TFs using protein domain and functional site information. The authors have also employed error-correcting output coding, a multi-class classification algorithm, in order to classify the identified TFs according to: basic-TFs, zinc-TFs, helix-TFs, and beta-TFs. These published works have demonstrated their accuracy and stability; however, there is no fully computational method developed to identify splicing factors based on protein sequences so far. Therefore, we are motivated to develop a novel method focusing on the identification of human splicing factors using the experimentally verified spliceosomal proteins and RNA-splicing related proteins.

In this study, the experimentally validated human splicing factors have been collected from two previously published literatures [14], [16]. This work not only investigates the composition of amino acids on splicing factors, but also considers evolutionary information through a position-specific scoring matrix (PSSM). The explored features are used to construct a predictive model for differentiating splicing factors from non-splicing proteins. A support vector machine (SVM) is used to construct a predictive model with various features. Moreover, the information of functional domains extracted from InterPro [41] is also adopted to improve the prediction scheme. Finally, an independent test set, which is not included in the training set, is also constructed to evaluate whether the predictive model is over-fitted to the training set.

## Materials and Methods

**Figure 1:**
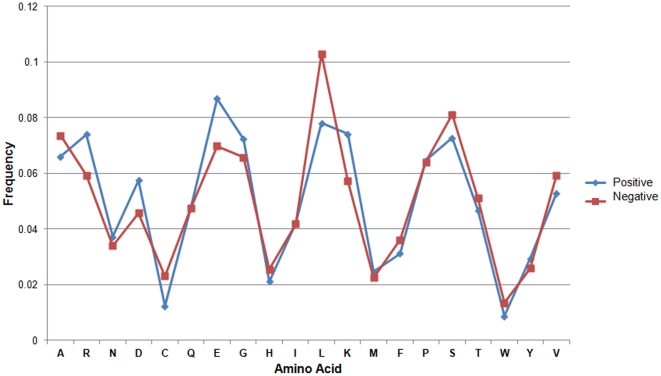
System Flow.

Figure 1 presents the system flow of the proposed method. It consists of the following steps: data collection and pre-processing, feature extraction, model learning and cross-validation, and independent testing. The details of each process are described as follows.

### Data collection and pre-processing

The experimentally verified splicing factors in humans were collected from published literatures [14], [16]. Jurica and Moore [14] have proposed about 180 manually curated splicing factors in humans by literature survey. In addition, Barbosa-Morais et al. [16] have proposed more than 200 splicing factors from multiple organisms by an integrative method incorporating systematic pipeline and experimental evidence. After the removal of redundant protein entries, it resulted in a total of 283 human splicing factors which are regarded as positive data for feature investigation and model training. Furthermore, human proteins which are not among the positive data obtained from literature were extracted from the UniProt protein knowledge base [42] by running a search on UniProt IDs using the keyword “HUMAN”. To construct the positive data of independent testing, only experimentally verified splicing factors are obtained from the resulting dataset by collecting protein entries annotated as “RNA splicing”, “spliceosome”, or “splicing factors”. UniProt uses such annotations to define a protein entry that has been experimentally identified to be essential for RNA splicing. This yielded 99 protein sequences which are then regarded as positive data for independent testing. In order to filter out potential noise data for non-splicing proteins, the remaining proteins consisting of keyword “RNA-binding” are removed. As a result, a total of 19512 proteins are regarded as negative data.

In classifying splicing factors and non-splicing proteins, there is a possibility that the prediction performance of the constructed models is overestimated due to an over-fit to the training set. Therefore, an independent test set is used to estimate the actual prediction performance. However, there may be a possible overestimation in the prediction performance due to homologous sequences found in the training data and independent test data. With reference to the work by Panwar et al. [43], homologous sequences from the collected data are removed by using CD-HIT. CD-HIT firstly forms a cluster with a representative sequence having the longest length which is then compared to the remaining sequences. If the similarity between a target sequence and the representative sequence is above the user-selected sequence identity threshold which refers to the pairwise sequence identity between two proteins, then the target sequence is considered homologous to the representative sequence [44]. Different values were tested for the sequence identity parameter as shown in Table 1. The resulting dataset given a sequence identity parameter of 30% contains 173 positive sequences of training set, 65 positive sequences of independent test set, and 11113 negative sequences. The negative data is then randomly divided into two sets – 5557 protein sequences are regarded as negative data for model training, and 5556 protein sequences are regarded as negative data for independent testing.

**Table 1:** Data abundancy after using CD-HIT.

Sequence identity	Positive data of training set	Positive data of independent test set	Negative data
100% (original)	283	99	19512
90%	274	94	18897
80%	268	94	18447
70%	256	94	17727
60%	242	88	16710
50%	226	82	15255
40%	202	80	13333
30%	173	65	11113

### Feature extraction

#### Compositions of amino acids and amino acid dipeptide

Each protein sequence in the data set is represented using a vector {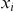, *i* = 1,…,*n*} labeled according to its corresponding protein group (e.g. splicing factor or non-splicing protein). The vector 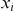 has 20 elements for the amino acid composition and 400 elements for the amino acid dipeptide composition. For amino acid composition, the 20 elements specify the numbers of occurrences of 20 amino acids normalized with the total number of residues in the protein. On the other hand, for amino acid dipeptide composition, the 400 elements specify the numbers of occurrences of 400 amino acid dipeptides normalized with the total number of dipeptides in the protein.

#### Statistically significant amino acid dipeptides

In further exploring potential features for protein classification, various methods aimed at selecting relevant sequence features given a large set of features have been used [45]. In this work, the importance of amino acid dipeptides in identifying splicing factors is further investigated by means of measuring the statistical significance of each dipeptide in the data set. For each amino acid dipeptide, the number of splicing factors and non-splicing proteins containing the target dipeptide is calculated separately. The statistical significance of each dipeptide is then obtained by examining a sample against a background set based on the hypergeometric equation (*P*-value) [46]:
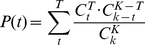
(1)where *K* is the background set represented by the number of all proteins and *T* is the sample set represented by the number of splicing factors; *k* is the number of all proteins having the target amino acid dipeptide and *t* is the number of splicing factors having the target amino acid dipeptide. *P*-value is calculated for each dipeptide based on the hypergeometric equation. A smaller *p*-value corresponds to a greater statistical significance. Furthermore, the positive and negative probabilities of each amino acid dipeptide are computed by means of dividing the number of splicing factors or non-splicing proteins having the target amino acid dipeptide by the total number of splicing factors or non-splicing proteins, respectively. The probability difference between the positive and the negative probability is then obtained. In this work, amino acid dipeptides having a *p*-value less than 0.05 and a probability difference greater than 0 is considered as statistically informative for the identification of splicing factors.

#### Evolutionary information

**Figure 2:**
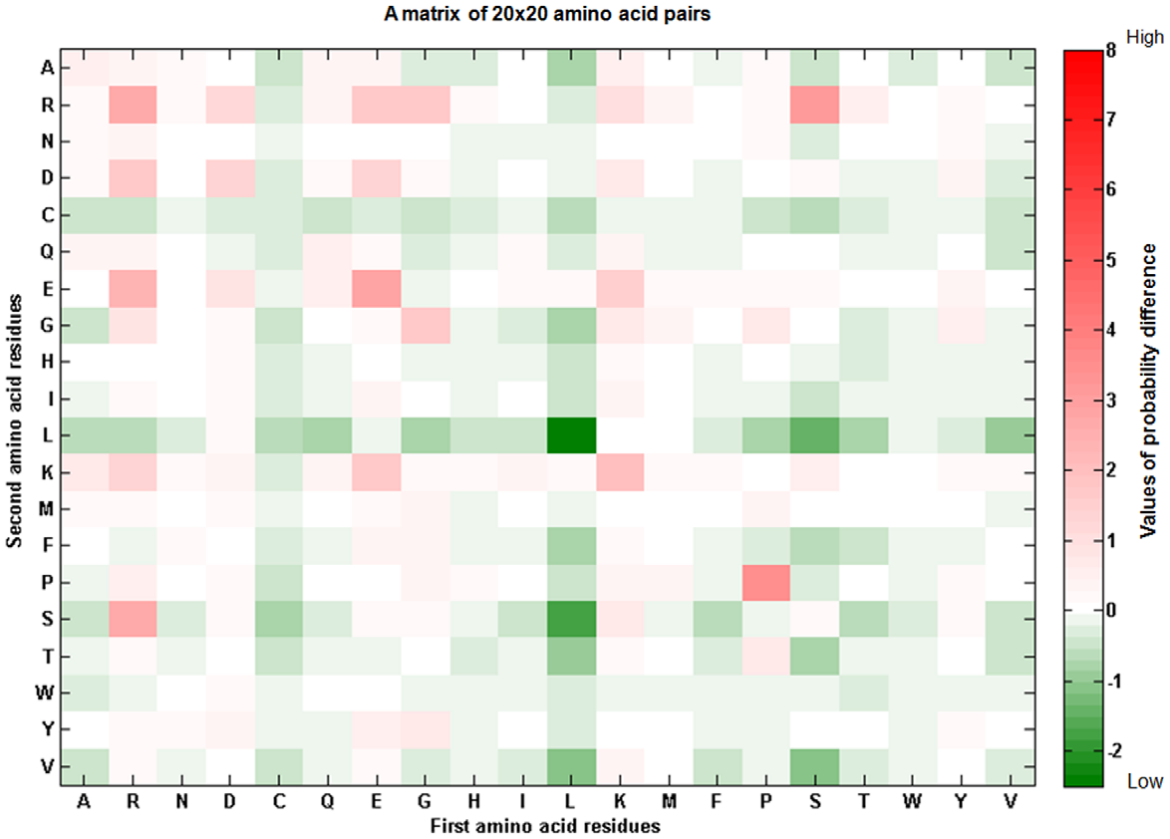
The detailed process of generating 400-dimensional PSSM vector by the PSSM profile.

Several amino acid residues of a protein can go through mutation without changing its structure, and two proteins may share similar structures with different amino acid compositions. In this work, evolutionary information is obtained using position-specific scoring matrix (PSSM). PSSM profiles have been extensively utilized in protein secondary structure prediction, subcellular localization and other approaches in bioinformatics [47], [48], [49]. The PSSM profiles of each protein were obtained by using PSI-BLAST search against the non-redundant database of protein sequences compiled by NCBI [50]. Due to the fact that the data consists of protein sequences with variable length, a weighted score of features is obtained by summing up the position-specific scores of the same amino acids occurring in a protein sequence to get a uniform number of features. Figure 2 displays in detail how to generate a 400-dimensional (20×20 residue pairs) PSSM feature vector for each splicing factor and non-splicing protein. PSSM profile is a matrix of *m*×20 elements where *m* represents the protein sequence length and 20 represents the position specific scores for each type of amino acid. Then, the PSSM profile is transformed to a 20×20 matrix by summing up each row of same amino acid in the PSSM profile and the variable is denoted as “x”. Finally, every element of 400-dimensional PSSM vector is divided by the length of the sequence and then is scaled by 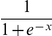 for normalizing the values between 0 and 1.

#### Information of functional domains

Previous works on protein prediction have exhibited the ability of distinguishable domain regions in the classification of proteins [45]. In this work, domain information is investigated as a feature for classifying splicing factors from non-splicing proteins. To investigate the preference of functional domains in splicing factors, this study referred to the annotations in InterPro [41]. InterPro is an integrated resource, which was developed initially as a means of rationalizing the complementary efforts of the PROSITE [51], PRINTS [52], Pfam [53], and ProDom [54] databases, for providing protein “signatures” such as protein families, domains and functional sites. The domain information of each splicing factor in the training data is collected by referring to its corresponding InterPro ID in the UniProt database. The collected domains are then analyzed in order to identify the most distinguishable domains in splicing factors. For this work, functional domains present in more than five splicing factors are considered as significant domains.

#### Feature Combination

A hybrid approach is investigated in this work by combining different sets of feature vectors with the goal of improving splicing factor prediction performance. Three types of hybrid combinations are explored. In the first combination, the effect of combining PSSM with the composition-based features is explored. In the second combination, the effect of combining domain information with the composition-based features is explored. In the third combination, the effect of combining both PSSM and domain information with the composition-based features is explored.

### Model learning and cross-validation evaluation

Support vector machine (SVM) is applied to generate computational models that incorporate the encoded set of features. Based on binary classification, the concept of SVM is to map the input samples into a higher dimensional space using a kernel function, and then to find a hyper-plane that discriminates between the two classes with maximal margin and minimal error. A public SVM library, LibSVM [55], is used to train the predictive model with positive and negative training sets, which are encoded with reference to various training features. The radial basis function (RBF) 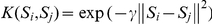 is selected as the kernel function of SVM. Cross-validation is important to the application of the predictor [56]. The predictive performance of the constructed models is evaluated by performing *k*-fold cross validation. The training data is divided into *k* groups by splitting each dataset into *k* approximately equal sized subgroups. In this work, *k* is set to five. During cross-validation, each subgroup is regarded as the validation set in turn, and the remainder is regarded as the training set. Next, the following measures of predictive performance of the trained models are defined:
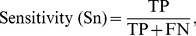
(2)

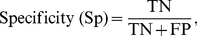
(3)

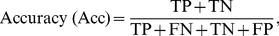
(4)where TP, TN, FP and FN represent the numbers of true positives, true negatives, false positives and false negatives, respectively. Additionally, the parameters of the predictive model, cost and gamma value of the SVM models are optimized to maximize predictive accuracy. In optimization of SVM parameter C and RBF kernel parameter gamma, the grid search is applied to obtain the parameters that achieve the best accuracy during k-fold cross-validation. Then, the hybrid combinations of features that yield the highest accuracy are employed to construct predictive models for independent testing. Finally, the SVM model trained with the combined features and the selected parameters (C and gamma) are evaluated the predictive performance using independent testing data.

### Independent testing

In order to further evaluate the trained models, an independent test set from humans is obtained as discussed previously, resulting in 65 positive data and 5556 negative data shown in Table 1. In addition, this work also investigates the ability of the predictive model to identify splicing factors from other mammalian species (File S1).

## Results and Discussion

### Investigation of amino acid composition in splicing factors

**Figure 3:**
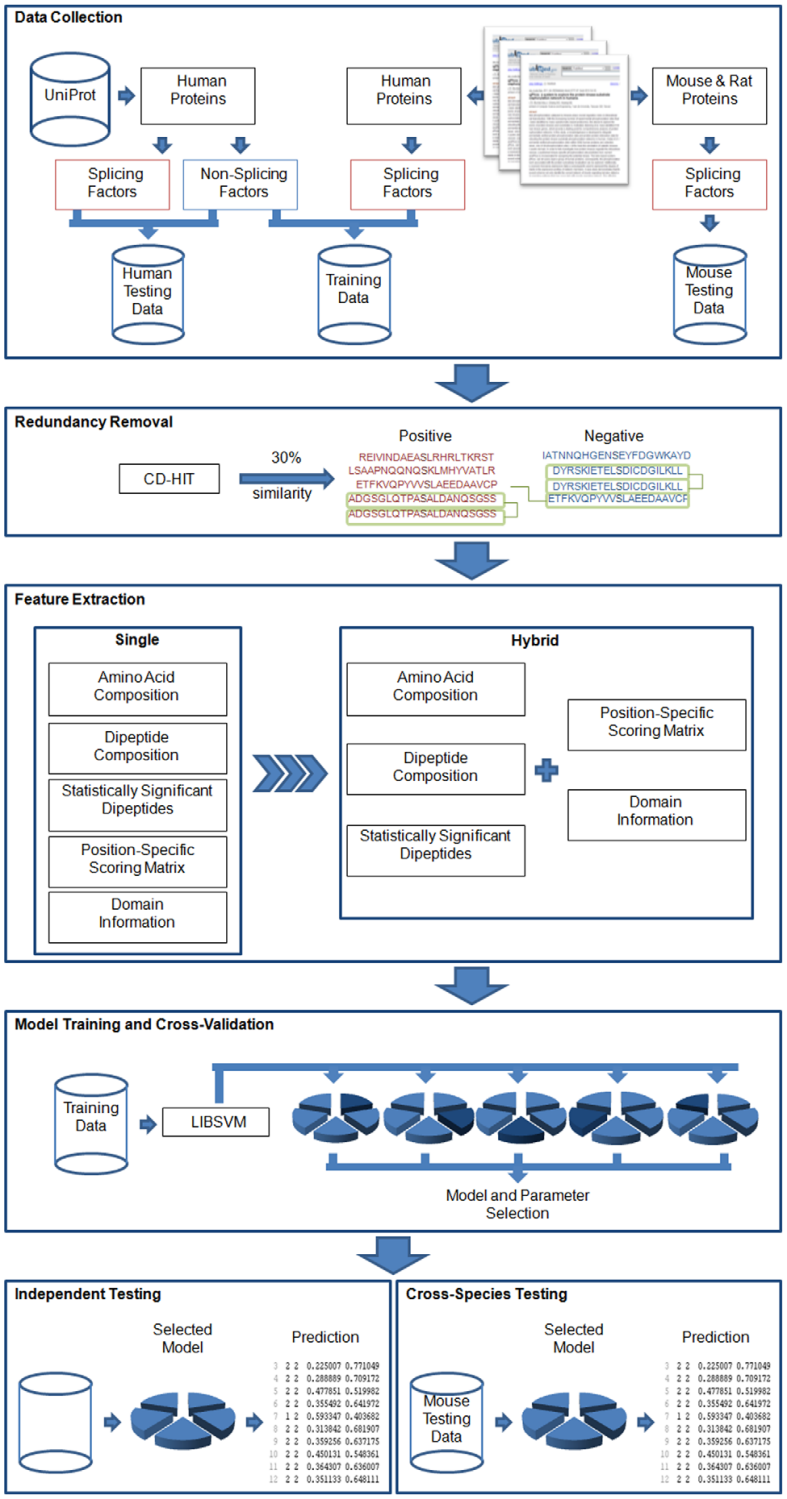
Percent composition of 20 amino acids between positive data (splicing factors) and negative data (non-splicing proteins).

The difference between splicing factors and non-splicing proteins is analyzed in terms of its amino acid composition as shown in Figure 3. It can be observed that splicing factors are significantly distinguishable from non-splicing proteins at the amino acid composition level. For instance, Arginine (R), Aspartic Acid (D), Glutamic Acid (E), Glycine (G), Leucine (L), and Lysine (K) residues all exhibit a remarkable difference between splicing factors and non-splicing proteins. The dominance of these amino acid residues indicates its contribution in RNA-protein and protein-protein interactions. Among these residues, the abundance of R and K in splicing factors is reasonable because these positively charged residules can easily interact with negatively charged RNA. Another abundant amino acid group observed in splicing factors is D and E which are negatively charged residues and are easily located on surface area of a protein for interacting with other splicing factors. Interestingly, the small size and flexibility of G residue is probably responsible for making it suitable for the structural adjustments required during the protein-protein interactions [37]. Furthermore, Leucine (L) is observed to be the most prominent among all under-representated residues. In order to examine the effectiveness of amino acid composition in identifying splicing factors, an SVM model is trained using a 20-dimensional vector consisting of the composition scores for twenty amino acids. The amino acid composition-based model is evaluated by means of five-fold cross-validation. As shown in Table 2, the model achieved 76.90% sensitivity, 80.33% specificity, and 80.22% accuracy.

**Table 2:** Five-fold cross validation performance of basic features.

Features	Sensitivity	Specificity	Accuracy
Amino acid composition	76.90%	80.33%	80.22%
Dipeptide composition	78.62%	78.53%	78.53%
Statistically significant dipeptides	76.31%	79.07%	78.98%
PSSM	79.81%	79.48%	79.49%
Functional domain	38.75%	93.82%	92.16%

### Investigation of amino acid dipeptide composition in splicing factors

Previous studies have exhibited that dipeptide composition-based methods can yield a better performance as compared to amino acid composition-based methods [43], [57]. In order to investigate this claim in terms of identifying splicing factors, an SVM model is trained using amino acid dipeptide composition as features. Firstly, the composition of all possible amino acid pairs is calculated in splicing factors and non-splicing proteins, respectively. Thus, each protein sequence can be encoded as a 400-dimensional vector consisting of the composition scores for 20×20 amino acid pairs. Using the resulting 400-dimensional dipeptide vectors, an SVM model is trained and is evaluated by means of five-fold cross-validation. The dipeptide composition-based model achieved 78.62% sensitivity, 78.53% specificity, and 78.53% accuracy as shown in Table 2. It can be observed that the amino acid composition-based method yields higher accuracy in identifying splicing factors. However, using dipeptide composition yields a more balanced sensitivity and specificity.

**Figure 4:**
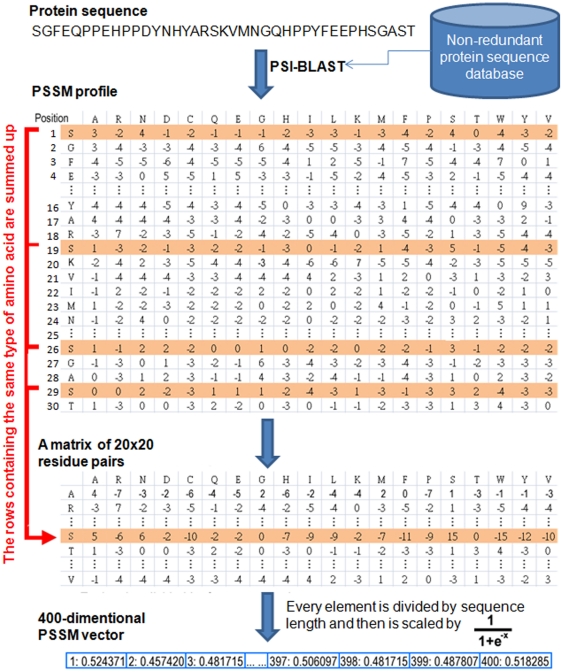
Probability difference of 20×20 amino acid pairs between splicing factors and non-splicing proteins. The amino acid pair with red box indicates an over-representation in splicing factors; on the other hand, green box means an under-representation.

The amino acid dipeptide composition of splicing factors and non-splicing proteins is further analyzed by means of selecting statistically significant dipeptides among the 400 amino acid pairs. Figure 4 shows the probability difference of 400 amino acid pairs between splicing factors and non-splicing proteins. In the 20×20 matrix, amino acid pairs marked in red indicates over-representation in splicing factors while amino acid pairs marked in blue indicates under-representation. It can be observed in Figure 4 that DD pairs are over-represented in splicing factors as well as D residues paired with E, R, and K. Also, KK pairs are observed to be over-represented in splicing factors. Furthermore, it can also be observed that Cysteine (C) residues paired with other resides are under-represented in splicing factors. The *P*-value and the probability difference of each amino acid dipeptide is calculated as discussed previously. After ranking the dipeptides according to *P*-value, each amino acid pair having a *P*-value<0.05 and a probability difference >0 is considered as a statistically significant pair. A total of 64 pairs are selected among the 400 amino acid pairs. Interestingly, it is found that these observations in Figure 4 coincide with the selected 64 significant pairs based on *P*-value (File S2).

An SVM model is trained using a 64-dimensional vector consisting of the composition scores for the selected 64 statistically significant amino acid dipeptides. The model is evaluated by means of five-fold cross-validation. As shown in Table 2, the statistically significant dipeptide-based model achieved 76.31% sensitivity, 79.07% specificity, and 78.98% accuracy. It can be concluded that the method used for selecting statistically significant dipeptides was able to select the features that mostly distinguish splicing factors from non-splicing proteins. Also, the method was able to maintain a performance similar to that yielded by using all 400 amino acid composition features. In line with this, it can be assumed that the dipeptides not selected by the method do not significantly distinguish splicing factors from non-splicing proteins.

### Investigation of evolutionary information

It has been shown in previous works that using evolutionary information encapsulated in a PSSM profile provides a more comprehensive information as compared to single sequence features [37]. In this work, the application of evolutionary information is investigated in terms of identifying splicing factors by training an SVM model using a 400-dimensional vector derived from the PSSM profile of each protein sequence. A PSSM profile is the probability of the occurrence of each type of amino acid residues at each position along with insertion/deletion. Hence, PSSM is regarded as a measure of residue conservation in a given protein sequence. As shown in Table 2, the PSSM-based model achieved 79.81% sensitivity, 79.48% specificity, and 79.49% accuracy.

### Investigation of functional domain information in splicing factors

In order to analyze functional domain information in splicing factors, the experimentally verified domains of each splicing factor in the training data is collected by referring to the “InterPro” field in UniProt. This resulted to a a total of 252 functional domains existing in splicing factors. In order to capture the representative functional domains in splicing factors, functional domains which are present in more than 5 splicing factors are selected as distinguishable domains. This resulted to 15 functional domains as shown in Table 3. It is observed that the most distinguishable functional domain is the “Nucleotide-bd a/b plait” with InterPro ID: IPR012677 which exists in 46 splicing factors. Another distinguishable functional domain is the “RRM” domain with InterPro ID: IPR000504 which exists in 45 splicing factors. In order to evaluate the performance of using the selected distinguishable domains, an SVM model is trained using a 15-dimensional vector consisting of the 15 distinguishable domains represented by a binary score: 1 if present and 0 otherwise. As shown in Table 2, the domain-based model achieved 38.75% sensitivity, 93.82% specificity, and 92.16% accuracy. It can be observed that using domain information alone is not sufficient to correctly identify all splicing factors as seen in the low sensitivity of the prediction model. As discussed previously, only those functional domains present in more than 5 splicing factors are considered by the model. This affected the prediction of true positives due to the fact that many splicing factors are not annotated with the selected functional domains. This may later improved given a more comprehensive InterPro annotation on the dataset. On the other hand, the high specificity yielded by the model signifies that the selected functional domains are meaningful since they do not exist in most of the non-splicing proteins.

**Table 3:** Statistics of InterPro functional annotations in 173 splicing factors.

InterPro ID	Description	Number of splicing factors
IPR012677	Nucleotide-bd a/b plait	46
IPR000504	RRM domain	45
IPR010920	LSM-related domain	11
IPR001163	LSM domain	11
IPR006649	LSM domain euk/arc	10
IPR015943	WD40/YVTN repeat-like domain	10
IPR001680	WD40 repeat	9
IPR011046	WD40 repeat-like domain	9
IPR019782	WD40 repeat 2	9
IPR017986	WD40 repeat domain	9
IPR019781	WD40 repeat sg	9
IPR015880	Znf C2H2-like	7
IPR019775	WD40 repeat CS	7
IPR020472	G-protein beta WD-40 repeat	6
IPR013083	Xnf RING/FYVE/PHD	6

InterPro classifies sequences at superfamily, family and subfamily levels and annotates the occurrence of functional domains, repeats and important sites. The annotations which occur in more than five splicing factors are presented with the information of InterPro ID, description, and number of splicing factors.

### Cross-validation performance using hybrid features

The composition-based features are combined with PSSM and domain information in order to investigate the effects of incorporating evolutionary information and domain information. Three types of hybrid combinations are explored in this study: the first type refers to the combination of basic sequence information with evolutionary information; the second type refers to the combination of basic sequence information with domain information; and the third type refers to the combination of basic sequence information with both evolutionary information and domain information. An SVM model is trained using each set of hybrid feature combination. As shown in Table 4, the amino acid composition-based model improved with 77.47% sensitivity, 82.94% specificity, and 81.77% accuracy when combined with the evolutionary information from PSSM profiles. Both dipeptide composition and statistically significant dipeptides-based models also improved with 79.81% sensitivity, 78.46% specificity, and 79.47% accuracy when combined with evolutionary information. With regard to incorporating basic features with domain information, the amino acid composition-based model yields a lower performance with 75.15% sensitivity, 82.94% specificity, and 82.04% accuracy. This is the same for the dipeptide composition-based model which yields 75.76% sensitivity, 77.34% specificity, and 77.29% accuracy. The statistically significant dipeptides-based model also yields a lower performance with 75.12% sensitivity, 76.96% specificity, and 76.91% accuracy. Furthermore, incorporating both domain and evolutionary information with statistically significant dipeptides gives the highest performance with 82.68% sensitivity, 81.78% specificity, and 81.81% accuracy. It is interesting to find that the three models converged at the same performance after incorporating both evolutionary and domain information.

**Table 4:** Five-fold cross-validation performance of hybrid features.

Hybrid features	Sensitivity	Specificity	Accuracy
**Incorporating PSSM with**			
Amino acid composition	77.47%	82.94%	81.77%
Dipeptide composition	79.81%	78.46%	79.47%
Statistically significant dipeptides	79.81%	78.46%	79.47%
**Incorporating Domain information with**			
Amino acid composition	75.15%	82.25%	82.04%
Dipeptide composition	75.76%	77.34%	77.29%
Statistically significant dipeptides	75.12%	76.96%	76.91%
**Incorporating both PSSM and Domain with**			
Amino acid composition	82.68%	81.77%	81.79%
Dipeptide composition	82.68%	81.77%	81.79%
Statistically significant dipeptides	82.68%	81.78%	81.81%

### Independent testing

The method is further evaluated by using an independent data set composed of collected human splicing factors and non-splicing proteins as discussed previously. The independent data is first tested on each model trained on single features as shown in Table 5. It can be observed that the amino acid composition-based model yields a lower performance with 68.07% sensitivity, 68.17% specificity, and 68.17% accuracy as compared to the models based on dipeptide composition and statistically significant dipeptides. The dipeptide composition-based model performs with 69.61% sensitivity, 69.64% specificity, and 69.63% accuracy while the statistically significant dipeptides-based model performs slightly higher with 69.61% sensitivity, 70.46% specificity, and 70.45% accuracy. With regard to the use of evolutionary information, the PSSM-based model achieved the highest performance among all single feature-based models with 72.69% sensitivity, 72.20% specificity, and 72.21% accuracy. On the other hand, similar to its cross-validation performance, the domain-based model performed with a low sensitivity of 21.53%, 93.63% specificity, and 92.79% accuracy.

**Table 5:** Predictive performance of basic features on an independent testing data.

Features	Sensitivity	Specificity	Accuracy
Amino acid composition	68.07%	68.17%	68.17%
Dipeptide composition	69.61%	69.64%	69.63%
Statistically significant dipeptides	69.61%	70.46%	70.45%
PSSM	72.69%	72.20%	72.21%
Functional domain	21.53%	93.63%	92.79%

The independent data is then tested on the models based on hybrid feature combinations. As presented in Table 6, the amino acid composition-based model improved in classifying the independent data with 72.69% sensitivity, 72.16% specificity, and 72.17% accuracy when combined with the evolutionary information from PSSM profiles. Both dipeptide composition and statistically significant dipeptides-based models also improved with 72.69% sensitivity, 72.20% specificity, and 72.21% accuracy when combined with evolutionary information. With regard to incorporating basic features with domain information, the amino acid composition-based model yields a slightly lower performance on the independent data with 68.07% sensitivity, 68.10% specificity, and 68.10% accuracy. The statistically significant dipeptides-based model also yields a lower performance with 66.53% sensitivity, 66.57% specificity, and 66.57% accuracy. On the other hand, the dipeptide composition-based model slightly improved with 68.07% sensitivity, 70.53% specificity, and 70.50% accuracy. Furthermore, incorporating both domain and evolutionary information to the basic feature-based models gives the highest performance with 74.23% sensitivity, 73.64% specificity, and 73.65% accuracy. Similar to the cross-validation performance, incorporating both domain information and evolutionary information on the three basic models allowed it to converge at the same prediction performance.

**Table 6:** Predictive performance of hybrid features on an independent testing data.

Hybrid features	Sensitivity	Specificity	Accuracy
**Incorporating PSSM with**			
Amino acid composition	72.69%	72.16%	72.17%
Dipeptide composition	72.69%	72.20%	72.21%
Statistically significant dipeptides	72.69%	72.20%	72.21%
**Incorporating Domain information with**			
Amino acid composition	68.07%	68.10%	68.10%
Dipeptide composition	68.07%	70.53%	70.50%
Statistically significant dipeptides	66.53%	66.57%	66.57%
**Incorporating both PSSM and Domain with**			
Amino acid composition	74.23%	73.10%	73.61%
Dipeptide composition	74.23%	73.64%	73.65%
Statistically significant dipeptides	74.23%	73.64%	73.65%

### Conclusion

Although the importance of splicing factors has been indicated in pre-mRNA splicing and alternatively splicing, *in vivo* or *in vitro* identification of splicing factors are subject to technical limitations. Here we propose a computational method to identify splicing factors on the basis of amino acid sequence of a protein. With reference to two previously published works, a total of 283 experimentally verified human splicing factors have been obtained in in this study. After the removal of homologous sequences, the investigation of amino acid composition reveals that there are remarkable differences between splicing factors and non-splicing proteins. The most prominent feature is the abundance of positively and negatively charged residues in splicing factors. Another important characteristic is the slight enrichment of G residues in splicing factors. A five-fold cross-validation evaluation has demonstrated that using amino acid composition could provide a promising prediction accuracy. Another basic feature, amino acid dipeptide composition, is also examined that has similar predictive performance to amino acid composition. Moreover, this method has presented that the evolutionary information could provide a balanced predictive performance, but the domain information resulted in low sensitivity and high specificity. However, the incorporation of evolutionary information and domain information improve the predictive performance compared to the models trained with basic features. Additionally, the independent testing has demonstrated that the constructed model can identify new splicing factors in human proteome, as well as in mouse and rat (File S1). Although several approaches have been proposed to computationally predict RNA-binding proteins [28], [29], these methods, such as the web server RNApred [28], provide a high sensitivity but a very low specificity using the collected human independent testing data.

The biological process of RNA splicing machinery has not yet been fully elucidated, partly because splicing factors are not yet exhaustively identified. The recent genome-wide sequencing techniques [16], [19], [26] provide an opportunity to exhaustively observe splicing factors in an organism. This work shows that the *in silico* identification could be a feasible means of conducting preliminary analyses as well as significantly reducing the number of potential targets that require further *in vivo* or *in vitro* confirmation.

## Supporting Information

File S1 **Cross-species Testing.**
(DOC)

File S2 **Dipeptide pair P-value.**
(XLSX)

## References

[1] Johnson JM, Castle J, Garrett-Engele P, Kan Z, Loerch PM (2003). Genome-wide survey of human alternative pre-mRNA splicing with exon junction microarrays.. Science.

[2] Chen L, Zheng S (2009). Studying alternative splicing regulatory networks through partial correlation analysis.. Genome Biol.

[3] Wang ET, Sandberg R, Luo S, Khrebtukova I, Zhang L (2008). Alternative isoform regulation in human tissue transcriptomes.. Nature.

[4] Keren H, Lev-Maor G, Ast G (2010). Alternative splicing and evolution: diversification, exon definition and function.. Nat Rev Genet.

[5] Maniatis T, Tasic B (2002). Alternative pre-mRNA splicing and proteome expansion in metazoans.. Nature.

[6] Smith CW, Valcarcel J (2000). Alternative pre-mRNA splicing: the logic of combinatorial control.. Trends Biochem Sci.

[7] Cartegni L, Chew SL, Krainer AR (2002). Listening to silence and understanding nonsense: exonic mutations that affect splicing.. Nat Rev Genet.

[8] Stamm S, Ben-Ari S, Rafalska I, Tang Y, Zhang Z (2005). Function of alternative splicing.. Gene.

[9] Matlin AJ, Clark F, Smith CW (2005). Understanding alternative splicing: towards a cellular code.. Nat Rev Mol Cell Biol.

[10] Stamm S (2008). Regulation of alternative splicing by reversible protein phosphorylation.. J Biol Chem.

[11] Wahl MC, Will CL, Luhrmann R (2009). The spliceosome: design principles of a dynamic RNP machine.. Cell.

[12] Corrionero A, Minana B, Valcarcel J (2011). Reduced fidelity of branch point recognition and alternative splicing induced by the anti-tumor drug spliceostatin A.. Genes Dev.

[13] David CJ, Manley JL (2010). Alternative pre-mRNA splicing regulation in cancer: pathways and programs unhinged.. Genes Dev.

[14] Jurica MS, Moore MJ (2003). Pre-mRNA splicing: awash in a sea of proteins.. Mol Cell.

[15] Zahler AM, Lane WS, Stolk JA, Roth MB (1992). SR proteins: a conserved family of pre-mRNA splicing factors.. Genes Dev.

[16] Barbosa-Morais NL, Carmo-Fonseca M, Aparicio S (2006). Systematic genome-wide annotation of spliceosomal proteins reveals differential gene family expansion.. Genome Res.

[17] Johnson PJ (2002). Spliceosomal introns in a deep-branching eukaryote: the splice of life.. Proc Natl Acad Sci U S A.

[18] Patel AA, Steitz JA (2003). Splicing double: insights from the second spliceosome.. Nat Rev Mol Cell Biol.

[19] Ben-Dov C, Hartmann B, Lundgren J, Valcarcel J (2008). Genome-wide analysis of alternative pre-mRNA splicing.. J Biol Chem.

[20] Black DL (2003). Mechanisms of alternative pre-messenger RNA splicing.. Annu Rev Biochem.

[21] Paz I, Akerman M, Dror I, Kosti I, Mandel-Gutfreund Y (2010). SFmap: a web server for motif analysis and prediction of splicing factor binding sites.. Nucleic Acids Res.

[22] Reed R (2000). Mechanisms of fidelity in pre-mRNA splicing.. Curr Opin Cell Biol.

[23] Neubauer G, King A, Rappsilber J, Calvio C, Watson M (1998). Mass spectrometry and EST-database searching allows characterization of the multi-protein spliceosome complex.. Nat Genet.

[24] Zhou Z, Licklider LJ, Gygi SP, Reed R (2002). Comprehensive proteomic analysis of the human spliceosome.. Nature.

[25] Rappsilber J, Ryder U, Lamond AI, Mann M (2002). Large-scale proteomic analysis of the human spliceosome.. Genome Res.

[26] Chen YI, Moore RE, Ge HY, Young MK, Lee TD (2007). Proteomic analysis of in vivo-assembled pre-mRNA splicing complexes expands the catalog of participating factors.. Nucleic Acids Res.

[27] Kasyapa CS, Kunapuli P, Cowell JK (2005). Mass spectroscopy identifies the splicing-associated proteins, PSF, hnRNP H3, hnRNP A2/B1, and TLS/FUS as interacting partners of the ZNF198 protein associated with rearrangement in myeloproliferative disease.. Exp Cell Res.

[28] Kumar M, Gromiha MM, Raghava GP (2010). SVM based prediction of RNA-binding proteins using binding residues and evolutionary information.. J Mol Recognit.

[29] Han LY, Cai CZ, Lo SL, Chung MC, Chen YZ (2004). Prediction of RNA-binding proteins from primary sequence by a support vector machine approach.. RNA.

[30] Ma X, Guo J, Wu J, Liu H, Yu J (2011). Prediction of RNA-binding residues in proteins from primary sequence using an enriched random forest model with a novel hybrid feature.. Proteins.

[31] Wang L, Huang C, Yang MQ, Yang JY (2010). BindN+ for accurate prediction of DNA and RNA-binding residues from protein sequence features.. BMC Syst Biol.

[32] Murakami Y, Spriggs RV, Nakamura H, Jones S (2010). PiRaNhA: a server for the computational prediction of RNA-binding residues in protein sequences.. Nucleic Acids Res.

[33] Liu ZP, Wu LY, Wang Y, Zhang XS, Chen L (2010). Prediction of protein-RNA binding sites by a random forest method with combined features.. Bioinformatics.

[34] Maetschke SR, Yuan Z (2009). Exploiting structural and topological information to improve prediction of RNA-protein binding sites.. BMC Bioinformatics.

[35] Wang Y, Xue Z, Shen G, Xu J (2008). PRINTR: prediction of RNA binding sites in proteins using SVM and profiles.. Amino Acids.

[36] Tong J, Jiang P, Lu ZH (2008). RISP: a web-based server for prediction of RNA-binding sites in proteins.. Comput Methods Programs Biomed.

[37] Kumar M, Gromiha MM, Raghava GP (2008). Prediction of RNA binding sites in a protein using SVM and PSSM profile.. Proteins.

[38] Wang L, Brown SJ (2006). Prediction of RNA-binding residues in protein sequences using support vector machines.. Conf Proc IEEE Eng Med Biol Soc.

[39] Terribilini M, Lee JH, Yan C, Jernigan RL, Honavar V (2006). Prediction of RNA binding sites in proteins from amino acid sequence.. RNA.

[40] Zheng G, Qian Z, Yang Q, Wei C, Xie L (2008). The combination approach of SVM and ECOC for powerful identification and classification of transcription factor.. BMC Bioinformatics.

[41] Hunter S, Apweiler R, Attwood TK, Bairoch A, Bateman A (2009). InterPro: the integrative protein signature database.. Nucleic Acids Res.

[42] Apweiler R, Bairoch A, Wu CH, Barker WC, Boeckmann B (2004). UniProt: the Universal Protein knowledgebase.. Nucleic Acids Res.

[43] Panwar B, Raghava GP (2010). Prediction and classification of aminoacyl tRNA synthetases using PROSITE domains.. BMC Genomics.

[44] Li W, Jaroszewski L, Godzik A (2001). Clustering of highly homologous sequences to reduce the size of large protein databases.. Bioinformatics.

[45] Wang L, Huang C, Yang JY (2011). Predicting siRNA potency with random forests and support vector machines.. BMC Genomics.

[46] Sadygov RG, Yates JR (2003). A hypergeometric probability model for protein identification and validation using tandem mass spectral data and protein sequence databases.. Anal Chem.

[47] Jones DT (1999). Protein secondary structure prediction based on position-specific scoring matrices.. Journal of Molecular Biology.

[48] Xie D, Li A, Wang MH, Fan ZW, Feng HQ (2005). LOCSVMPSI: a web server for subcellular localization of eukaryotic proteins using SVM and profile of PSI-BLAST.. Nucleic Acids Research.

[49] Ou YY, Gromiha MM, Chen SA, Suwa M (2008). TMBETADISC-RBF: Discrimination of beta-barrel membrane proteins using RBF networks and PSSM profiles.. Computational Biology and Chemistry.

[50] Altschul SF, Madden TL, Schaffer AA, Zhang J, Zhang Z (1997). Gapped BLAST and PSI-BLAST: a new generation of protein database search programs.. Nucleic Acids Res.

[51] Bairoch A (1991). PROSITE: a dictionary of sites and patterns in proteins.. Nucleic Acids Res.

[52] Attwood TK, Beck ME, Bleasby AJ, Parry-Smith DJ (1994). PRINTS–a database of protein motif fingerprints.. Nucleic Acids Res.

[53] Sonnhammer EL, Eddy SR, Durbin R (1997). Pfam: a comprehensive database of protein domain families based on seed alignments.. Proteins.

[54] Corpet F, Gouzy J, Kahn D (1998). The ProDom database of protein domain families.. Nucleic Acids Res.

[55] Chang C-C, Lin C-J (2001). LIBSVM : a library for support vector machines.. http://www.csie.ntu.edu.tw/~cjlin/libsvm.

[56] Chou KC, Shen HB (2007). Recent progress in protein subcellular location prediction.. Anal Biochem.

[57] Bhasin M, Raghava GP (2004). Classification of nuclear receptors based on amino acid composition and dipeptide composition.. J Biol Chem.

