# Supplementary material for: Incorporating Evolutionary Information and Functional Domains for Identifying RNA Splicing Factors in Humans
Source: PLoS One. 2011 Nov 16;6(11):e27567. doi: 10.1371/journal.pone.0027567 (PMC3217973; doi:10.1371/journal.pone.0027567)
Supplement: File S1 — Cross-species Testing. (DOC) [file pone.0027567.s001.doc]

**Cross-species Testing**

In order to test the ability of the models to identify splicing factors from other mammalian species, experimentally verified splicing factors in mouse and rat species were collected from published literature [1]. The resulting 143 protein sequences are regarded as positive data for cross-species testing. Given that this is not the major focus of this work; a negative data set is not procured for this testing phase. Similar to the processing of the training data and independent data sets, homologous sequences in the collected cross-species data (mouse and rat) are also removed by using CD-HIT with a sequence identity parameter of 30%. This resulted to 66 mouse and rat splicing factors which are used to further test the trained models with regard to cross-species prediction performance. The cross-species independent data is tested on the best performing model according to cross-validation and independent testing performance. The test shows that the models trained with human data yield a sensitivity of 97.20% on mouse and rat data.

**References**

1. Barbosa-Morais NL, Carmo-Fonseca M, Aparicio S (2006) Systematic genome-wide annotation of spliceosomal proteins reveals differential gene family expansion. Genome Res 16: 66-77.
